# Physical and Mechanical Properties of Thermally-Modified Beech Wood Impregnated with Silver Nano-Suspension and Their Relationship with the Crystallinity of Cellulose

**DOI:** 10.3390/polym11101538

**Published:** 2019-09-20

**Authors:** Siavash Bayani, Hamid R. Taghiyari, Antonios N. Papadopoulos

**Affiliations:** 1 Department of Wood and Paper Science and Technology, College of Agriculture and Natural Resources, Science and Research Branch, Islamic Azad University, Tehran, Iran; 2 Wood Science and Technology Department, Faculty of Materials Engineering & New Technologies, Shahid Rajaee Teacher Training University, Tehran, Iran; 3 Laboratory of Wood Chemistry and Technology, Department of Forestry and Natural Environment, International Hellenic University, GR-661 00 Drama, Greece

**Keywords:** thermal modification, nanocompounds, mechanical and physical properties, cellulose, crystallinity

## Abstract

The aim of this study was to investigate the physical and mechanical properties of thermally modified beech wood impregnated with silver nano-suspension and to examine their relationship with the crystallinity of cellulose. Specimens were impregnated with a 400 ppm nanosilver suspension (NS); at least, 90% of silver nano-particles ranged between 20 and 100 nano-meters. Heat treatment took place in a laboratory oven at three temperatures, namely 145, 165, and 185 °C. Physical properties and mechanical properties of treated wood demonstrated statistically insignificant fluctuations at low temperatures compared to control specimens. On the other hand, an increase of temperature to 185 °C had a significant effect on all properties. Physical properties (volumetric swelling and water absorption) and mechanical properties (MOR and MOE) of treated wood demonstrated statistically insignificant fluctuations at low temperatures compared to control specimens. This degradation ultimately resulted in significant decrease in MOR, impact strength, and physical properties. However, thermal modification at 185 °C did not seem to cause significant fluctuations in MOE and compression strength parallel to grain. As a consequence of the thermal modification, part of amorphous cellulose was changed to crystalline cellulose. At low temperatures an increased crystallinity caused some of the properties to be improved. Crystallinity also demonstrated a decrease in NS-HT185 in comparison to HT185 treatment. TCr indices in specimens thermally treated at 145 °C revealed a significant increase as a result of impregnation with nanosilver suspension. This improvement in TCr index resulted in a noticeable increase in MOR and MOE values. Other properties did not show significant fluctuations, suggesting that the effect of the increased crystallinity and cross-linking in lignin was more than the negative effect of the low cell-wall polymer degradation caused by thermal modification. Change of amorphous cellulose to crystalline cellulose, as well as cross-linking in lignin, partially ameliorated the negative effects of thermal degradation at higher temperatures and therefore, compression parallel to grain and modulus of elasticity did not decrease significantly. Overall, it can be concluded that increased crystallinity and cross-linking in lignin can compensate for some decreased properties caused by thermal modification, but it would be significantly dependent on the temperature under which modification is carried out. Impregnating specimens with silver nano-suspension prior to thermal modification enhanced the effects of thermal modification as a result of improved thermal conductivity.

## 1. Introduction

The main drawbacks of wood, namely dimensional instability and biological durability are mainly due to the nature of the cell wall main polymers and in particular due to their high abundance of hydroxyl groups (OH) [1,2,3]. The protection of wood without toxic chemicals has been the subject of numerous studies. The application of chemical or thermal modification technique can be considered as a solution to this, since species of inferior properties, mainly softwoods, can be modified and transformed to entirely new green products with superior properties [4,5,6]. An interesting article, reviewed recently the state of the art in the area of chemical and thermal modification. The article was focused on the two most promised techniques, namely acetylation and furfurylation [7].

Though thermal modification has been successful in improving dimensional stability and resistance against fungal attack [8,9], it reduces mechanical properties in wood. The reduction in mechanical properties limits the industrial applications in which the strength of wood is of prime importance. It is to be kept in mind that there is always a trade-off between the improved dimensional stability and fungal resistance versus the reduced mechanical properties. Degradation of main wood components (cell-wall polymers including cellulose, hemicellulose, and lignin) caused by thermal modification is a well-known phenomenon [3]. The outcome of this degradation is usually materialized by increased weight loss as the temperature for thermal modification increases. However, under relatively mild thermal conditions (<200 °C), some semi-crystalline cellulose regions change to crystalline regions [10] and cross-linking and polycondensation reactions in lignin structure occur [11,12]. These can have significant improving impact on different properties of wood, though weight loss may show very little degradation of cell-wall polymers.

Nanotechnology, an attractive science, seems to have a remarkable potential to create products of a new generation with enhanced properties [13]. The change in material properties is primarily due to the large interfacial area that is developed per unit of volume, since the level of added particles is reduced to nanometers. Nanomaterials enhance the properties of the original material, shows great compatibility with the traditional materials and cause limited alteration of their original features [14,15]. Their use in wood has the objective to improve its physical and mechanical properties and its durability against microorganisms, since it is generally acceptable that nanosized metals and minerals interact with the bacterial elements, leading gradually to the cell death [14,15,16,17] or even to the disruption of the enzyme function [15,18]. Moreover, wood has a low thermal conductivity [14] and therefore, it is expected that the outer layer and inner part of individual specimens would not be similarly modified by thermal modification. Impregnating specimens with a nano-metal suspension, such as silver, would improve the thermal conductivity of specimens, eventually making both outer and inner parts to be thermally modified to nearly same degree [19,20]. The cited authors studied physical properties (water absorption and thickness swelling), and mechanical properties (MOR, MOE, compression parallel to grain, brittleness, and pull-off strength), however, little or no studies were carried out on the effects of thermal modification at different temperatures on crystallinity and cellulose content of nanosilver-impregnated specimens. 

The sustainability, excellent mechanical properties and interesting physical properties of cellulose nanomaterials have recently received a great deal of attention for modifying polymers and acting as both active and passive components in a wide range of potential products. The applications for which cellulose nanomaterials have been investigated include medical and pharmaceutical applications, paper and paperboard additives, paints and coatings, security papers, food packaging, electronic devices and displays, and energy harvesting and storage devices [21,22,23].

As cellulose is broken down into nanometer-scaled particles and fibers, the materials begin to exhibit some interesting and unique properties depending on their size, morphology, charge, and other characteristics [23,24]. Typically, cellulose nanomaterials, especially from plant sources, can be categorized as either cellulose nanocrystals (CNCs) or cellulose nanofibrils (CNFs). CNCs are discrete, rod-shaped cellulose particles typically with high crystallinity and are primarily produced through acid hydrolysis of native cellulose. CNCs have nanometer-scaled diameters with lengths of hun dreds to thousands of nanometers, and they often contain surface charge groups and can thus be colloidally stable [24,25,26]. Conversely, CNFs are typically network structured nanoscaled fibers that can be produced from countless methods usually involving some kind of chemical treatment followed by mechanical refining. The morphology of CNFs widely varies from process to process, and they can often have a wide distribution of diameters and lengths [26,27,28].

Cellulose nanomaterials are often touted as potential polymer reinforcements because of their high strength and stiffness, and these good mechanical properties also lend these materials for use in functional devices such as electronics. The cellulose crystal has been measured to have extremely high strength and stiffness with values of about 10 GPa and up to about 200 GPa, respectively, but bulk materials, such as films, made from nanocellulose have considerably lower mechanical properties [28,29,30]. Nonetheless, cellulose nanomaterial films and composites have mechanical properties that rival or exceed many polymers. The optical properties of cellulose nanomaterials are also distinctive from bulk cellulose and can be favorable for electronic applications. Cellulosic materials have low thermal expansion, which is desirable in electronic components that generate heat, and the coefficient of thermal expansion (CTE) for cellulose nanomaterials and their compos ites has been measured and reported for various CNF films [31,32]. Because of all these properties, including sustainability, high strength, flexibility, transparency, and low thermal expansion, cellulose nanomaterials have been explored for their use in electronics and functional devices. In some cases, cellulose nanomaterials can provide advantages over traditional materials [26,27,28,30,32]. The aim of this study, therefore, was to investigate the physical and mechanical properties of thermally modified beech wood impregnated with silver nano-suspension and to examine their relationship with the crystallinity of cellulose.

## 2. Materials and Methods

### 2.1. Sample Preparation

Beech trees (*Fagus orientalis*) were cut from Shafaroud district at the Rezvanshahr city in Gilan province (Iran). One-meter log from each tree was cut at the breast height to be air-dried for more than five months. From each log, twenty-one specimens for each of the properties studied in the present project were prepared; these specimens were randomly divided into seven groups of control, heat-treated (HT) at 145, 165, and 185 °C, as well as nanosilver-impregnated heat-treated (NS-HT) at the same temperatures. The chemical composition of beech wood, as far as the main cell wall polymers is concerned, is as follows: 69% holocellulose, 47.6% cellulose, and 25.5% lignin. Once impregnated, all specimens were again air-dried for three more months to the final moisture content of 8% before any test was carried out on them, because wood has a thermo-hygromechanical behavior and its properties depend on the combined action of temperature, relative humidity, and mechanical load variations [33].

### 2.2. Nanosilver Impregnation

In this study, silver nano-suspension was applied due to its easy of application and its effectiveness in increasing thermal conductivity in wood and wood-composites [14,19,20]. Specimens were impregnated with a 400 ppm nanosilver suspension (NS). At least 90% of silver nano-particles ranged between 20 and 100 nano-meters. A pressure of 2.5 bars for 10 minutes was applied in a sealed tank. Once impregnated, the specimens were randomly placed under the same conditions (30 °C, 40%–45% relative humidity) along with the un-treated specimens for two months. For checking the depth of impregnation, separate specimens were prepared to be simultaneously impregnated with NS specimens. After the impregnation, these specimens were cut in half to observe that NS-suspension has penetrated in the core section of specimens. 

### 2.3. Thermal Modification

**Figure 1:**
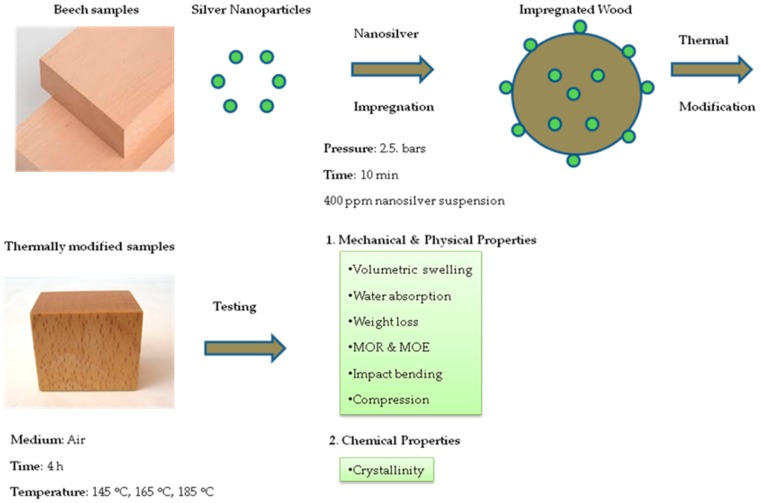
Flow diagram of the experimental procedure.

Specimens were heat-treated in a laboratory oven (model Memmert UFE 700), filled with atmospheric air. Un-treated and nanosilver-impregnated specimens were randomly arranged on the middle trays. Wooden strips of 3-mm thickness were put under specimens to avoid direct contact with metal trays of the oven. For HT-145 and NS-HT-145 specimens, thermal treatment was carried out at 145 °C for 24 h. For HT-165 and NS-HT-165 specimens, thermal treatment was carried out at two stages; the first stage was heating at 145 °C for 24 h continued with heating at 165 °C for 4 h. The flow diagram of the experimental procedure is depicted in Figure 1.

### 2.4. Total Crystillanity Index (TCrI)

Fourier Transform Infrared Spectroscopy (FTIR) was used to measure crystallinity of cellulose in the present project. In this method, specific infrared bands in FTIR spectra are related to the crystallinity and the type of cellulose (amorphous or crystalline) in each specimen [34]. The total crystallinity index (TCrI or TCI) was used to measure and report the amount of crystallinity in different treatments. In this index, the band (at 1372 cm^−1^) is attributed to C–H deformation, and the band at 2900 cm^−1^ is attributed to C–H and CH_2_ stretching. The areas under the curve of the above-mentioned bands were measured using OMNIC 6.1a software (Thermo Nicolet Corporation, USA). The areas were measured based on the on-set and off-set points for each band. Once the area under each band was measured, TCr index for each treatment was defined and calculated as a_1372cm_^−1^ divided by a_2900cm_^−1^ [34]. It is to be noted that a linear relationship was found between the results from TCrI with those obtained from X-ray diffraction (XRD) [35]. 

### 2.5. Physical and Mechanical Properties

Physical and mechanical properties, namely volumetric swelling, water absorption, modulus of rupture (MOR), modulus of elasticity (MOE), impact bending, compression strength parallel to grain, were determined in accordance with ASTM D0143-94 [36].

### 2.6. Statistical Analysis

Statistical analysis was conducted using SAS software, version 9.2 (Cary, NC, USA). Two-way analysis of variance (ANOVA) was performed on the mean data to determine significant differences at the 95% level of confidence. Duncan’s multiple range groupings were carried out among treatments to elucidate significant difference among groups. Hierarchical cluster analysis, including dendrograms and Ward methods with squared Euclidean distance intervals, was conducted with SPSS/18, version 18 (IBM; NY, USA). Cluster analysis was performed to find similarities and dissimilarities between treatments based on more than one property simultaneously. The scaled indicator in each cluster analysis shows similarities and differences between treatments; lower scale numbers show more similarities while higher ones show dissimilarities. Fitted-line, contour, and surface plots were done in Minitab software, version 16.2.2 (Minitab Inc.; NY, USA). 

## 3. Results and Discussion

**Figure 2:**
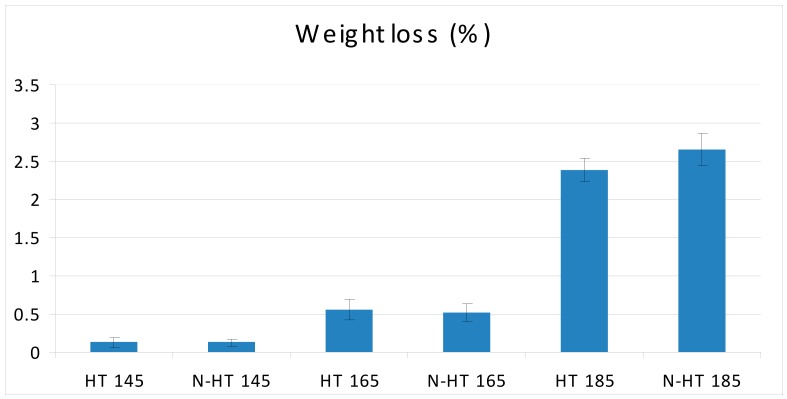
Weight losses in beech wood heat-treated at 145, 165, and 185 °C and impregnated with silver nano-suspension (HT = heat treatment at a determined temperature; N = nanosilver impregnated).

Results showed an increasing trend in weight losses as the temperature of thermal modification increased from 145 °C to 185 °C (Figure 2). The highest and lowest weight losses were observed in NS-HT185 and HT145 specimens, respectively. Weight losses caused by thermal modification at 145 °C, were very low, both for untreated and NS-impregnated specimens. This indicated that degradation of cell-wall polymers was very low at this temperature. It is expected that mostly hemi-cellulose and lignin were degraded at this temperature as to the fact that cellulose tends to have higher resistance to thermal degradation in comparison to hemi-cellulose and lignin [11,12,37].

**Figure 3:**
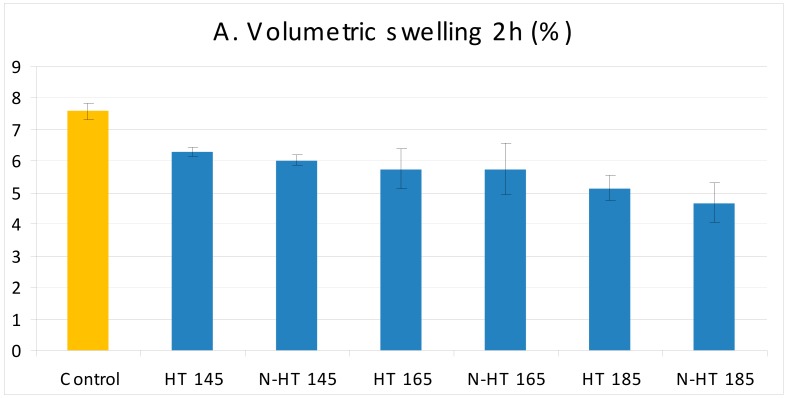

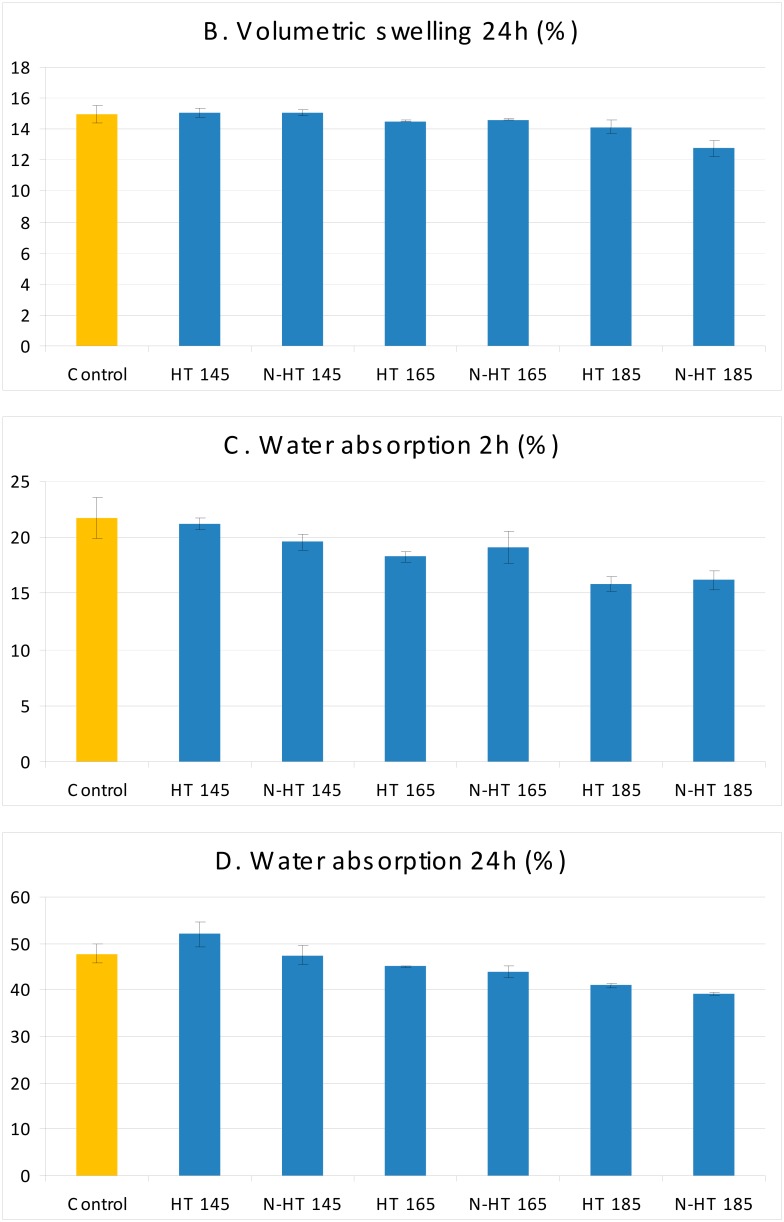
Physical properties in beech wood heat-treated at 145, 165, and 185 °C and impregnated with silver nano-suspension (HT = heat treatment at a determined temperature; N = nanosilver impregnated).

**Figure 4:**
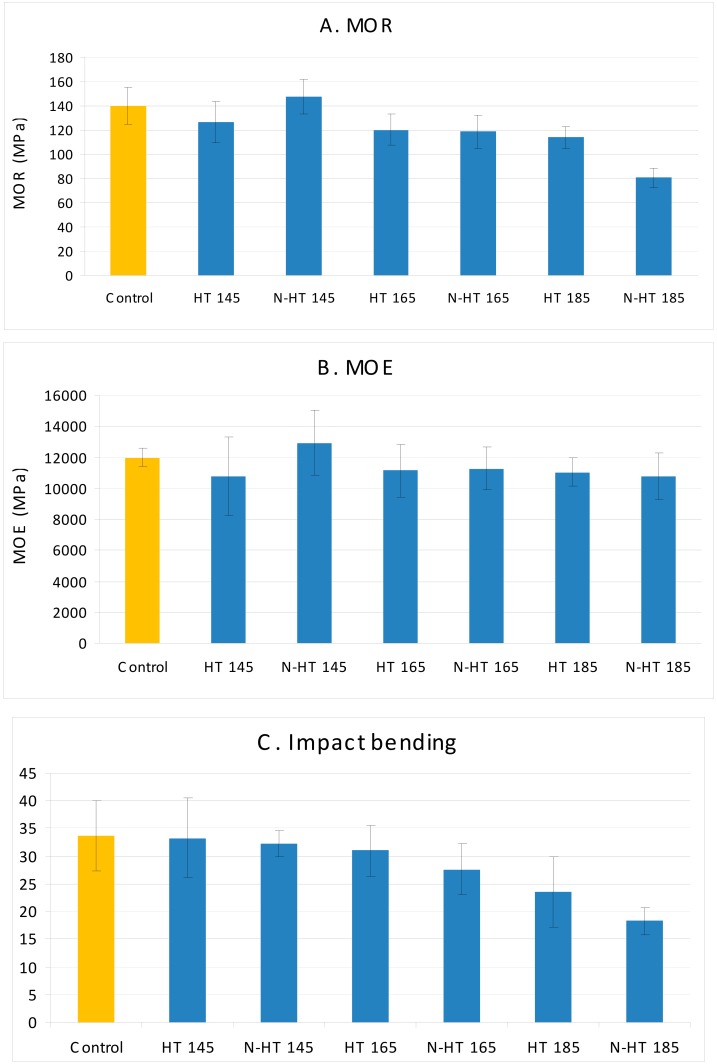

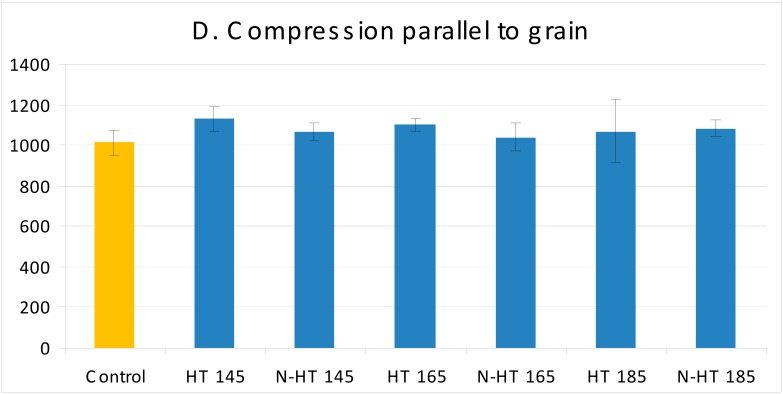
Mechanical properties in beech wood heat-treated at 145, 165, and 185 °C and impregnated with silver nano-suspension (HT=Heat Treatment at a determined temperature; N = nanosilver impregnated).

**Figure 5:**
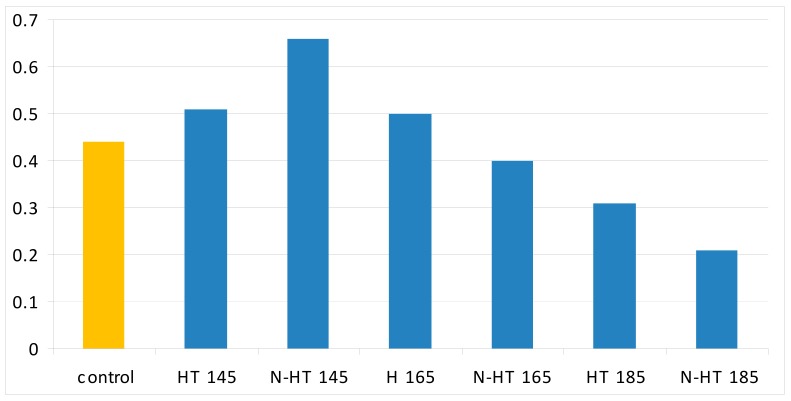
Crystallinity in the seven treatments studied in the present project (HT = heat treatment at a determined temperature; N = nanosilver impregnated).

Physical properties (volumetric swelling and water absorption) (Figure 3A–D) and mechanical properties (MOR and MOE) (Figure 4A,B) of treated wood demonstrated statistically insignificant fluctuations at low temperatures compared to control specimens. These are considered corroborating evidence of low degradation of cell-wall polymers at this low temperature of 145 °C for thermal modification. On the other hand, an increase of temperature to 185 °C had a significant effect on all properties. There was a significant weight loss, indicating degradation in cell-wall polymers. This degradation ultimately resulted in significant decrease in MOR, impact strength, and physical properties. However, thermal modification at 185 °C did not seem to cause significant fluctuations in MOE and compression strength parallel to grain (Figure 4B,D). In fact, compression strength parallel to grain showed an increase, though the increase was not statistically significant. In this connection, it was reported that some semi-crystalline regions in wood changes to crystalline regions as a result of heat treatment [38]. The crystalline regions in wood structure act as a framework that supports the whole wood structure and therefore, increase in this part had an improving effect on MOE and compression strength parallel to grain values, though cell-wall polymers were degraded to some extent. However, crystallinity measurement showed a decrease when specimens were thermally modified at 185 °C (Figure 5) and therefore, other factors appeared to be involved in the process too. Thermal modification in the present study was carried out at three temperatures which are all more than glass transition of lignin in most wood species [3,38,39]. Therefore as a binding agent, cross-linking in lignin could occur, making new bonds and connecting points between lignin with cellulose and hemicellulose fibrils that were thermally degraded [10]. This reasoning can also partly explain the increases in MOR and MOE values in N-HT145 specimens, though the increase was not statistically significant (Figure 4A,B). In fact, the increased crystallinity as well as cross-linking in lignin put the small thermal degradation in perspective, eventually MOR and MOE increased. That is, these could significantly ameliorate the negative effects of thermal degradation. Attenuated Total Reflectance (ATR) graph of crystallinity was in agreement with this hypothesis, showing the highest crystallinity in N-HT145 treatment (Figure 5). Water absorption and volumetric swelling values also illustrated significant decreases when specimens were heat-treated at 185 °C, indicating thermal degradation of cellulose and hemicellulose, change of amorphous cellulose to crystalline cellulose, and cross-linking in lignin binding cellulose molecules and inhibiting their hydroxyl groups to make bonds with water molecules. Moreover, the reduction in the physical properties can also be partially attributed to irreversible hydrogen bonding in the course of water movements within the pore system of the cell walls, eventually decreasing hygroscopicity in wood [40]. As to the fact that one of the consequences of irreversible hydrogen bonding is reported to be hornification, the unexpected high values of compression strength parallel to grain and MOE in HT185 and NS-HT185 treatments can also partially be attributed to hornification. 

**Figure 6:**
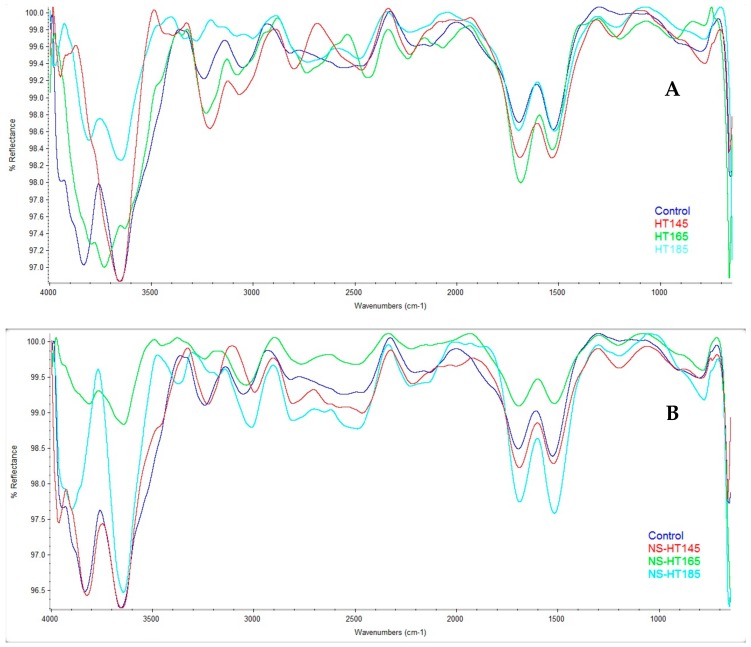
Infrared spectra of the control and thermally-modified (**A**) and the control and nanosilver suspension (NS)-impregnated thermally modified (**B**) beech specimens (HT = heat treatment at a determined temperature; NS = nanosilver impregnated).

Infrared spectra were in agreement with the above-mentioned changes in physical and mechanical properties as a result of thermal modification at different temperatures (Figure 6 A,B). Clear fluctuations and differences were observed in wave numbers 3300–3000 cm^−1^, relating to hydroxyl groups of cell-wall polymers which are greatly influential on both physical and mechanical properties. Significant differences were also observed in the intensities of fingerprint regions of wave number 1700–1500 cm^−1^ (C=O stretching) in both un-impregnated and NS-impregnated treatments. The peak of 1733 cm^−1^ was reduced in intensity in almost all thermally modified specimens; only NS-HT165 showed an increased intensity. This wave number is assigned to the stretching vibrations of carbonyl groups which belong mainly to hemicellulose. The wave number 3700–3600 cm^−1^ (relating to nonbonded hydroxy groups, OH stretch) showed similarity between the control and specimens heat-treated at 145 °C, both nanosilver-impregnated and untreated. Specimens heat-treated at 165 and 185 illustrated significant differences with that of control specimens, showing the effects of thermal modification on hydroxy groups. 

Previous studies, reported increased thermal conductivity in solid wood and wood-based composites with addition of nano-metals and nano-minerals [41,42]. Physical and mechanical properties of composite panels were improved as a result of addition of nano-copper in particleboards [41,42]; and an increased thermal conductivity of 29% by addition of nano-wollastonite was associated with improved physical and mechanical properties [42]. In the present study, thermal conductivity coefficient was not measured however, the effects of impregnating specimens with nanosilver suspension were observed in some of the properties. Weight loss in NS-HT185 specimens was higher, though the amount of increase was not statistically significant. This had a decreasing effect on volumetric swelling, MOR, and compression strength parallel to grain. Crystallinity also demonstrated a decrease in NS-HT185 in comparison to HT185 treatment. TCr indices in specimens thermally treated at 145 °C revealed a significant increase as a result of impregnation with nanosilver suspension. This improvement in TCr index resulted in a noticeable increase in MOR and MOE values. Other properties did not show significant fluctuations, suggesting that the effect of the increased crystallinity and cross-linking in lignin was more than the negative effect of the low cell-wall polymer degradation caused by thermal modification. The same hypothesis concurs with NS-HT165 and HT165 treatments. That is, no significant difference was observed between these two treatments on the basis of any of the properties, with the exception of impact bending. Impact bending (impact strength) was previously reported to be one the most sensitive property in solid wood species to be affected by thermal modification [10].

**Figure 7:**
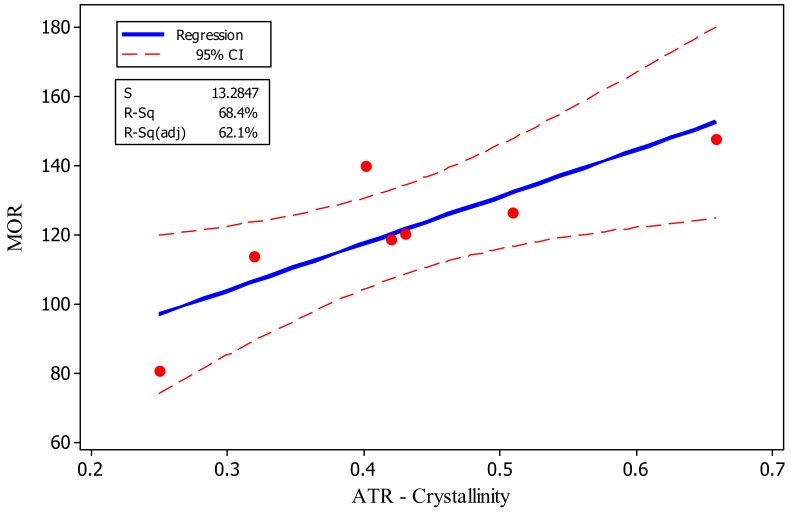
Fitted-line plot between Attenuated Total Reflectance (ATR)-crystallinity versus mechanical properties (MOR) in the seven treatments studied in the present project.

**Figure 8:**
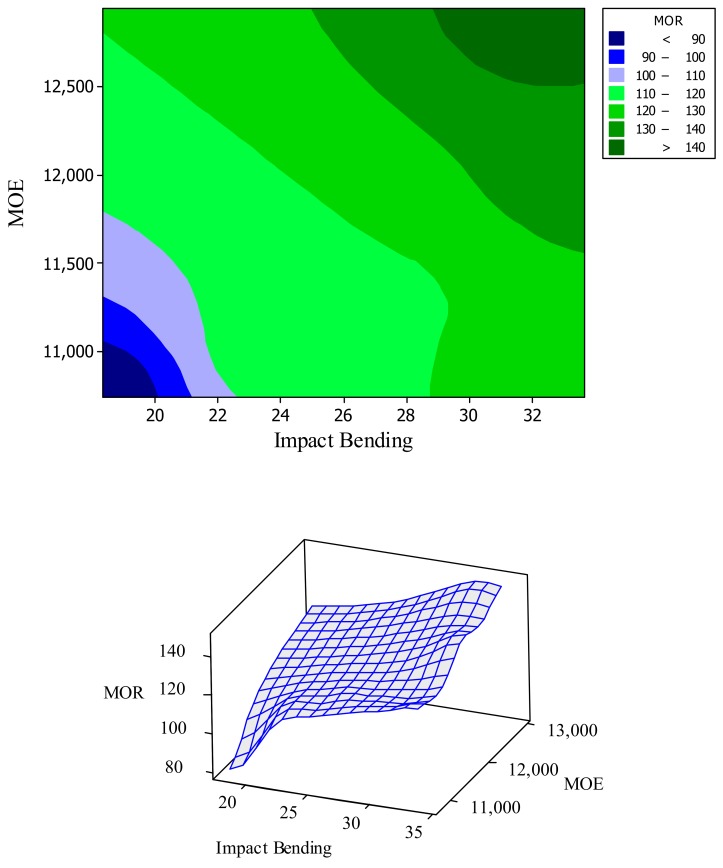
Contour and surface plots among MOR, MOE (mechanical properties), and impact bending in the seven treatments studied in the present project.

Fitted-line plot between different properties versus crystallinity showed rather low and statistically insignificant R-square. For instance, R-square between MOR versus ATR-crystallinity was only 68% (Figure 7). This implied the interaction and involvement of a variety of factors besides crystallinity. For example, and with regard to the physical properties of water absorption and volumetric swelling, degradation of cell-wall polymers and change in lignin may have caused alteration of chemical structure of the polymers and therefore, the number of hydroxyl groups to absorb water molecules differed. As to the mechanical properties, degradation of cell-wall polymers had negative effects on most of the mechanical properties, while increased crystallinity acted as a supporting framework, eventually ameliorating part of the negative effects. Overall, it can be concluded that increased crystallinity and cross-linking in lignin can compensate for some decreased properties caused by thermal modification, but it would be significantly dependent on the temperature under which modification is carried out. Contour plot and surface analysis illustrated the same trends. That is, a general direct and smooth relationship was observed among some of the properties, like MOR and MOE versus impact bending (Figure 8), indicating that thermal modification had the same increasing or decreasing effect on all these properties. However, distortions happened in the plots for some other properties, indicating significant different effect of thermal modification on the properties. 

**Figure 9:**
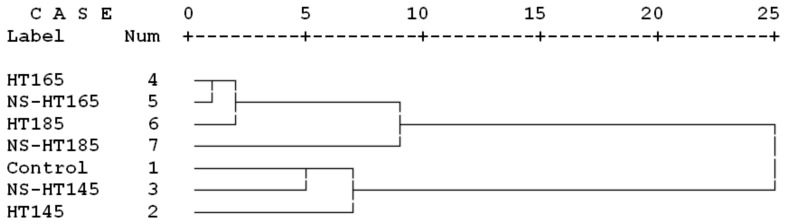
Cluster analysis based on all physical and mechanical properties, as well as crystallinity values of the seven treatments studied in the present project (HT = heat treatment at a determined temperature; NS = nanosilver impregnated).

Cluster analysis based on all properties measured in the present project showed rather close clustering of control, HT145, and NS-HT145 treatments (Figure 9). This indicated that the overall alterations of different properties caused by thermal modification at 145 °C only slightly changed the overall properties of beech wood, though some of properties revealed improvements, such as MOR and MOE. The other four treatments at 165 °C and 185 °C temperatures showed distinct remote clustering from the control treatment, demonstrating a great impact on the overall properties of beech wood. NS-HT185 clustered rather remotely from HT165, NS-HT165, and HT185 which were closely clustered. This can indicate the influential effect of an increased thermal conductivity caused by impregnation with NS-suspension. However, close clustering of HT146 with NS-HT145 can indicate that at lower temperature of about 145 °C, an increased thermal conductivity may not have as great impact in small woody specimens as it has at higher temperature of 185 °C. 

## 4. Conclusions

The aim of this study was to investigate the physical and mechanical properties of thermally modified beech wood impregnated with silver nano-suspension and to examine their relationship with the crystallinity of cellulose. Specimens were impregnated with a 400 ppm nanosilver suspension (NS); at least, 90% of silver nano-particles ranged between 20 and 100 nano-meters. Heat treatment took place in a laboratory oven at three temperatures, namely 145, 165, and 185 °C. Physical properties and mechanical properties of treated wood demonstrated statistically insignificant fluctuations at low temperatures compared to control specimens. On the other hand, an increase of temperature to 185 °C had a significant effect on all properties. As a consequence of the thermal modification, part of amorphous cellulose was changed to crystalline cellulose. At low temperatures an increased crystallinity caused some of the properties to be improved. Change of amorphous cellulose to crystalline cellulose, as well as cross-linking in lignin, partially ameliorated the negative effects of thermal degradation at higher temperatures and therefore, compression parallel to grain and modulus of elasticity did not decrease significantly. Previous studies, reported increased thermal conductivity in solid wood and wood-based composites with addition of nano-metals and nano-minerals [14,19,20]. The increased thermal conductivity ultimately caused higher degradation in the main wood components and therefore, mechanical properties and pull-off strength decreased in specimens thermally modified at temperature higher than 165 °C [19,20]. In the present study, thermal conductivity coefficient was not measured however, the consequent effects of impregnating specimens with nanosilver suspension were observed in some of the properties. Impregnating specimens with silver nano-suspension prior to thermal modification enhanced the effects of thermal modification as a result of improved thermal conductivity.
